# Gut microbiota and autism spectrum disorder: advances in dietary intervention strategies based on the microbiota-gut-brain axis mechanism

**DOI:** 10.3389/fnins.2025.1587818

**Published:** 2025-06-04

**Authors:** Ziji Fang, Yinxiong Zhou, Ke Chen, Juelan Wang, Xiaoli Liu, Ping Jia

**Affiliations:** ^1^School of Medicine, University of Electronic Science and Technology of China, Chengdu, China; ^2^Department of Nutrition, Chengdu Women’s and Children’s Central Hospital, School of Medicine, University of Electronic Science and Technology of China, Chengdu, China; ^3^Sichuan Provincial People’s Hospital, School of Medicine, University of Electronic Science and Technology of China, Chengdu, China; ^4^People’s Hospital of Deyang City, Deyang, China; ^5^Department of Neurosurgery, Sichuan Provincial People’s Hospital, University of Electronic Science and Technology of China, Chengdu, China

**Keywords:** autism spectrum disorder, gut-brain axis, gut microbiota, dietary intervention strategies, microbiota-metabolism axis

## Abstract

Autism spectrum disorder (ASD) is a complex neurodevelopmental disorder that is primarily characterized by deficits in social interaction, impaired communication, restricted interests, and repetitive behaviors. The prevalence of ASD has been steadily increasing, establishing it as one of the leading causes of disability among children worldwide. Although the precise pathogenesis of ASD remains unclear, factors such as genetic predisposition, environmental exposures, immune dysregulation, and neurodevelopmental abnormalities are collectively believed to contribute to its onset. In recent years, the gut microbiota has emerged as a promising area of research in neurobiology, particularly in relation to advances in understanding the microbiota–gut–brain axis (MGBA) mechanism. Studies have shown that children with ASD exhibit significant dysbiosis in their gut microbiota, which may affect brain function via the MGBA, ultimately leading to abnormal behaviors and impaired emotional regulation. This review summarizes the role of the gut microbiota in the pathogenesis of ASD, examining how alterations in gut permeability, dysregulated microbial metabolites, and immune dysfunction may influence ASD symptomatology. In particular, the role of the MGBA in modulating immune-inflammatory responses, neurodevelopment, and behavioral regulation has become a focal point of ASD research. Building on this foundation, the review further summarizes dietary intervention strategies grounded in the MGBA theory, emphasizing their potential to restore gut microbial composition, modulate immune responses, and enhance metabolic function, thereby offering novel therapeutic perspectives for ASD.

## 1 Introduction

ASD is a complex neurodevelopmental disorder that is characterized by deficits in social interaction, impaired communication, restricted interests, and repetitive behaviors (First, 2013). The prevalence of ASD has been steadily increasing, establishing it as one of the leading causes of disability among children worldwide. Statistics indicate that approximately 1% of children worldwide are affected by ASD (Zeidan et al., 2022). In China, the prevalence among children aged 0–6 years is 1.8% (Zhou et al., 2020), and 2020 data from the United States reveal that 2.76% of 8-year-olds have ASD, representing an increase of 0.46% compared to 2018 (Maenner et al., 2021; Maenner et al., 2023). Although the precise pathogenesis of ASD remains unclear, research indicates that its etiology involves a combination of genetic factors, environmental exposures, immune dysregulation, and neurodevelopmental abnormalitiess (Guerra et al., 2024; Masini et al., 2020; Zhang et al., 2019). In recent years, with rapid advancements in microbiome research, the gut microbiota has emerged as a promising research frontier in neurobiology (Kiani, 2023; Tremlett et al., 2017). The gut microbiota constitutes a complex ecosystem comprising bacteria, fungi, viruses, and other microorganisms, with bacteria playing a dominant role (Milani et al., 2017). These microorganisms are not only essential for digestion and nutrient absorption but also engage in bidirectional communication with the central nervous system via endocrine, metabolic, and immune pathways—a complex network referred to as the MGBA (Cryan et al., 2019). Studies have demonstrated that the gut microbiota composition in children with ASD differs markedly from that of healthy children, and such dysbiosis may affect brain function via the MGBA, ultimately leading to behavioral abnormalities and impaired emotional regulation (Alharthi et al., 2022; Chen et al., 2020; De Sales-Millán et al., 2024; Ding et al., 2020). Moreover, gastrointestinal dysfunction in children with ASD is closely associated with the severity of their core symptoms, further suggesting that the MGBA may play a key role in the pathophysiology of ASD (Meguid et al., 2022). Although numerous studies have revealed an association between gut microbiota dysbiosis and ASD, the precise mechanisms underpinning this relationship remain unclear. For instance, questions remain regarding how microbial metabolites influence neurodevelopment and the role of immune-inflammatory responses within the MGBA. This review aims to explore the relationship between the gut microbiota and ASD, elucidate the role of the MGBA in ASD pathogenesis, and summarize dietary intervention strategies grounded in this mechanism. By integrating current research findings, this review seeks to provide novel directions and strategies for future ASD research and therapy, thereby advancing the field toward more precise, individualized interventions.

## 2 Gut microbiota characteristics in children with ASD

Studies have shown that there is a close association between ASD and gut microbiota dysbiosis (Su et al., 2024). The underlying mechanisms involve alterations in intestinal permeability, abnormal microbial metabolites, the actions of neuroactive compounds, and regulation via the immune mediated MGBA. Children with ASD commonly exhibit increased intestinal permeability—often referred to as “leaky gut”—which allows microbial metabolites, such as short-chain fatty acids (SCFAs) and neuroactive compounds (e.g., 5-HT, GABA), to enter systemic circulation (Ullah et al., 2024). This, in turn, may disrupt the hypothalamic-pituitary-adrenal (HPA) axis, leading to elevated cortisol levels and contributing to anxiety and repetitive behaviors. In terms of the composition of the gut microbiota, children with ASD exhibit significant abnormalities at the phylum level. Specifically, the proportion of Firmicutes is markedly reduced, the Bacteroidetes-to-Firmicutes ratio is decreased, and the abundance of Acidobacteria is increased (Dan et al., 2020; Li et al., 2024; Soltysova et al., 2022). Butyrate-producing bacterial groups—such as *Ruminococcaceae*, the Eubacterium rectale group, *Lachnospiraceae*, and *Erysipelotrichaceae*—are reduced in children with ASD, while the abundance of *Clostridium* is significantly increased (Alshammari et al., 2020; Liu et al., 2019; Zhao et al., 2019). Recent studies with improved cohort homogeneity have further validated characteristic microbial biomarkers of ASD. For instance, the abundance of *Bifidobacterium and Prevotella* is reduced, whereas *Veillonella parvula*, *Lactobacillus rhamnosus*, and *Desulfovibrio* are enriched in the gut of individuals with ASD (Karnachuk et al., 2021; Zhang et al., 2018; Zhang et al., 2020). Notably, conflicting findings have been reported regarding the abundance of certain bacterial taxa (e.g., *Megasphaera* and *Barnesiella*), potentially due to population heterogeneity, methodological variability, or confounding factors such as constipation and allergies (Averina et al., 2020; De Sales-Millán et al., 2024; Zhao et al., 2019; Zou et al., 2020). Findings regarding alpha and beta diversity in the gut microbiota of individuals with ASD remain inconsistent, with some studies failing to detect significant differences (Wei-lan et al., 2020). However, genus-level alterations appear to be more consistent. For example, the abundance of *Sutterella* and *Desulfovibrio* is increased in the gut of children with ASD; the latter may produce hydrogen sulfide through sulfate reduction, potentially exacerbating intestinal inflammation and contributing to stereotyped behaviors (Dargenio et al., 2023; Heberling and Dhurjati, 2015; Lewandowska-Pietruszka et al., 2023). The reduction in *Bifidobacterium* not only impairs the synthesis of SCFAs but is also associated with decreased serum levels of folate and vitamin B12. Studies have shown that children with ASD exhibit significantly lower serum levels of folate and vitamin B12 compared to healthy controls, and that the metabolism of these vitamins depends on the biosynthetic activity of the gut microbiota (LeBlanc et al., 2017; Rudzki et al., 2021; Yektaş et al., 2019). Moreover, the monotonous dietary habits commonly observed in individuals with ASD—such as a preference for high-sugar foods and inadequate intake of fruits and vegetables—further exacerbate vitamin deficiencies and microbial dysbiosis, thereby creating a vicious cycle (Canals-Sans et al., 2022; Esteban-Figuerola et al., 2019; Raspini et al., 2021). Although numerous studies support an association between gut microbiota dysbiosis and ASD, the reproducibility of these findings across different cohorts remains limited—particularly in alpha and beta diversity analyses, which frequently yield contradictory results. While gut microbiota dysbiosis may contribute to the pathophysiology of ASD, the precise mechanisms by which this occurs remain unclear. Future studies should be grounded in the MGBA framework to investigate in greater depth how the gut microbiota influences neurotransmitter function, immune responses, and microbial metabolite production, thereby contributing to ASD pathogenesis.

## 3 The role of MGBA and neuroinflammation in ASD pathophysiology

The MGBA is composed of complex interactions among the gut microbiota, the mucosal immune system, the enteric nervous system, the autonomic nervous system, and the central nervous system, which receives input primarily through the vagus nerve (Ullah et al., 2023). This axis plays a critical role in the pathophysiology of ASD. Recent studies have increasingly shown that individuals with ASD commonly exhibit dysfunction of the autonomic nervous system, particularly characterized by parasympathetic dysregulation and reduced vagal tone (Muscatello et al., 2023; Xiao et al., 2023). These autonomic abnormalities are closely associated with core ASD symptoms, including deficits in social interaction and language impairments (Kong et al., 2023; Villar de Araujo et al., 2023). The vagus nerve, serving as a key communication channel between the gut and the brain, regulates parasympathetic activity, inflammatory responses, and MGBA signaling. Through these regulatory functions, it exerts substantial influence on ASD symptoms and comorbidities such as anxiety and gastrointestinal dysfunction (Chiu et al., 2016; Panju et al., 2015). Notably, microbiota-targeted precision therapies have been shown to significantly improve social deficits in ASD mouse models; however, this therapeutic effect is abolished following vagotomy, indicating that the gut microbiota’s influence on behavior is mediated via vagal signaling (Sgritta et al., 2019). This finding highlights the vagus nerve’s integral role not only in neural regulation but also in conveying the effects of gut-derived metabolites, such as SCFAs, to the brain, thereby impacting synaptic plasticity and neuroinflammation. Furthermore, microbial metabolites, such as SCFAs, can be conveyed to the central nervous system via the vagus nerve, thereby affecting synaptic plasticity and neuroinflammation (Needham et al., 2021). Neuroinflammation is a hallmark feature of ASD. Pro-inflammatory cytokines, including IL-1β and IL-6, can impair brain development by disrupting synaptic plasticity, synaptogenesis, and neurotransmitter regulation (Benarroch, 2024; Khairova et al., 2009; Levin and Godukhin, 2017; Madore et al., 2016). Activation of microglia and increased expression of glial genes observed in ASD patients further support the role of neuroinflammation in ASD pathon important role in the immune-inflammatory mechanisms of ASD. Alterations in the gut microgenesis (Lampiasi et al., 2023; Liao et al., 2020). Inflammatory dysregulation is evident in both peripheral and central systems. In peripheral blood, ASD patients often present with elevated white blood cell counts, increased monocyte levels, and widened platelet distribution width, indicating ongoing systemic inflammation (Ferencova et al., 2023; Harutyunyan et al., 2021). These peripheral pro-inflammatory cytokines, including IL-8 and TNF-α, are also elevated in brain tissue and cerebrospinal fluid, suggesting that systemic inflammation may influence central nervous system (CNS) function either through blood-brain barrier (BBB) leakage or active transport (Matta et al., 2019; Prata et al., 2017). BBB dysfunction is considered a key mechanism through which peripheral inflammation contributes to CNS pathology in ASD, allowing inflammatory molecules and immune cells to infiltrate neural tissue and exacerbate neuroinflammation (Candelario-Jalil et al., 2022; Cervellati et al., 2020; Galea, 2021). Furthermore, dysregulation of the MGBA further exacerbates this process: alterations in the gut microbiota can lead to increased intestinal permeability, triggering systemic inflammation and oxidative stress, which subsequently affect brain function. (Dumitrescu et al., 2018; Sochocka et al., 2019).

**FIGURE 1:**
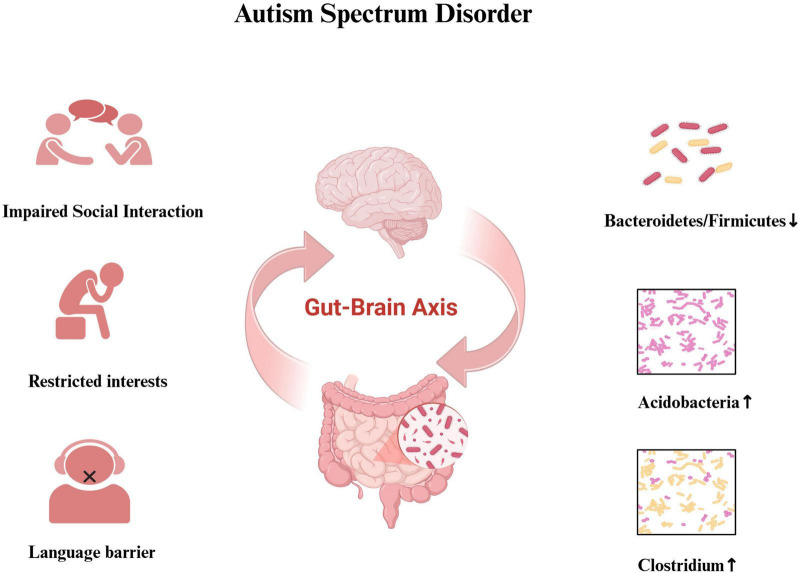
Intestinal microecological imbalance and ASD symptoms.

Although a substantial body of research supports the association between ASD and immune-inflammatory dysregulation, the exact mechanisms—particularly those varying by sex and age—remain to be fully elucidated. These findings underscore the importance of continued investigation. Future research should prioritize the diagnostic and therapeutic potential of immune-inflammatory biomarkers in ASD, and explore precision treatments targeting specific cytokines or inflammatory pathways, paving the way for personalized medical approaches (Figure 1).

## 4 Role and perspectives of dietary interventions in the management of ASD

Dietary interventions for ASD encompass a range of strategies designed to improve symptoms in children with ASD by modifying dietary patterns, eliminating potential allergens, or supplementing specific nutrients. These approaches may act through multiple pathways—such as modulating the gut microbiota, regulating immune responses, and enhancing metabolic functions—thus offering novel therapeutic avenues for ASD (Amadi et al., 2022). A 12-month randomized controlled trial conducted by Adams et al. demonstrated that comprehensive nutritional and dietary interventions can significantly improve the nutritional status, nonverbal IQ, and autism symptoms in children with ASD (Adams et al., 2018). However, study results vary greatly across populations. Factors such as age, sex, baseline diet, medication use, and regional microbiome differences can confound findings (Johnson and Howell, 2021). Likewise, geographically distinct cohorts show different microbiome profiles even after accounting for ASD diagnosis (Fouquier et al., 2021). These observations underscore the heterogeneity of ASD and its microbiome, and suggest that apparent microbiota changes in diet studies may in part reflect uncontrolled variables (Li et al., 2023). Consequently, many reported diet-related improvements should be interpreted with caution.

### 4.1 Gluten-free and casein-free diets

**FIGURE 2:**
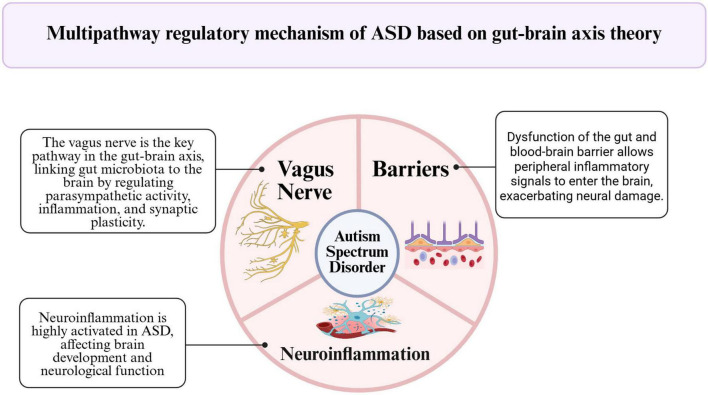
The critical role of the gut-brain axis in the pathogenesis of ASD.

The gluten-free and casein-free (GFCF) diet is among the most widely implemented nutritional interventions for ASD, based on the hypothesis that opioid-like peptides derived from gluten and casein may affect brain function (Önal et al., 2023). The theoretical basis for the GFCF diet originates from observations of abnormal immune responses to gluten and casein in children with ASD (Bjørklund et al., 2024; Długosz et al., 2025). Damage to the intestinal barrier is hypothesized to allow large molecules, such as gluten and casein, to enter the bloodstream, thereby triggering the release of pro-inflammatory cytokines (e.g., IL-6 and TNF-α), which may adversely affect neural function through the opioid system. Numerous small-scale studies have reported behavioral improvements following GFCF diets (Ghalichi et al., 2016; Quan et al., 2022); however, these findings are often constrained by methodological limitations. Early investigations were frequently open-label or non-randomized, introducing a high risk of bias (Whiteley et al., 2013). Even in randomized controlled trials (RCTs), adequate blinding has proven difficult, and sample sizes have generally been small (Elder et al., 2006; Lucarelli et al., 1995). Most current research suggests that the efficacy of the GFCF diet on core autism symptoms remains inconclusive. Notely, systematic reviews have failed to demonstrate consistent or sustained benefits (Keller et al., 2021). In summary, although some trials have reported improvements with GFCF regimens, the overall evidence remains mixed and limited by small sample sizes and insufficient methodological rigor. Observed improvements may also be influenced by placebo effects or concurrent nutritional changes. Given that many children with ASD already exhibit selective eating patterns, dietary changes may alter overall nutrient intake or reduce exposure to additive-rich foods, rather than specifically implicating gluten or casein. Therefore, rigorously controlled trials with sufficient blinding and larger cohorts are needed to determine whether GFCF diets have a genuine impact on ASD symptoms (Figure 2).

### 4.2 Probiotic and prebiotic modulation

Probiotics and prebiotics, as key modulators of the gut microbiota, have shown potential in ameliorating symptoms of ASD (Liu et al., 2022; Sanlier and Kocabas, 2023; Ullah et al., 2025; Yahfoufi et al., 2018). Studies indicate that probiotics can increase the abundance of beneficial bacteria, inhibit the growth of pathogens, and promote the production of SCFAs—thereby enhancing immune function (Chakravarty et al., 2025; Quigley and Quera, 2006; Salminen and Isolauri, 2006). However, most pediatric trials to date have been small, open-label, and lacked long-term follow-up; they also did not fully control for dietary habits, medication use, age, or geographic differences, all of which can markedly influence microbiota compositio. Mechanistic studies further propose that increased intestinal permeability (“leaky gut”) may allow bacterial metabolites and endotoxins—such as lipopolysaccharide—to enter the circulation and activate systemic and neuroinflammatory pathways (Doenyas, 2018; Duque et al., 2021). Yet whether this mechanism operates in children with ASD has not been directly tested in large, controlled clinical settings.

In a Chd8 deficient mouse model, excessive epithelial proliferation and microvillus abnormalities were linked to BBB dysfunction and microglial activation; probiotic treatment significantly suppressed these neuroinflammatory pathways (Ji et al., 2025). CHD8 represents one genetic ASD subtype and may not generalize to idiopathic cases; moreover, mice and humans differ in microbiota, diet, and environmental exposures (e.g., fiber intake, antibiotic history), so translation to ASD children remains speculative.

Moreover, SCFAs and tryptophan metabolites, as key mediators of bidirectional gut-brain communication, play essential roles in maintaining intestinal barrier integrity, modulating immune function, and transmitting signals via the vagus nerve (Dalile et al., 2019; Pan et al., 2025). In children with ASD, a reduction in butyrate-producing bacteria with an enrichment of propionate producers may imbalance SCFA ratios, potentially affecting synaptic plasticity and neurotransmitter homeostasis. It remains unclear whether SCFA shifts precede behavioral deficits or arise secondary to ASD-associated changes. Probiotic supplementation can upregulate butyrate and modulate dopamine D2 receptor expression, improving social deficits in mouse models (Chen et al., 2024; Miao et al., 2024; Mintál et al., 2022; Sanikhani et al., 2020).

Prebiotics enhance probiotic proliferation and restore gut microbiota balance, which may alleviate gastrointestinal symptoms in ASD patients (Duque et al., 2021; Grimaldi et al., 2018; Palmer et al., 2025). The fermentative properties of prebiotics can promote the proliferation of specific beneficial bacteria, leading to the production of metabolites such as butyrate. These metabolites, by activating G protein-coupled receptors (GPR41/43) on intestinal epithelial cells, further enhance barrier integrity and suppress the NF-kB signaling pathway, thereby mitigating systemic inflammation (Siddiqui and Cresci, 2021). While several double-blind RCTs demonstrate therapeutic potential particularly in reducing hyperactivity, irritability, and enhancing social functionings (Grimaldi et al., 2018; Voigt et al., 2024), other trials fail to show benefits on core ASD symptoms (He et al., 2023; Mazzone et al., 2024). This discrepancy appears attributable to methodological variations in trial duration, blinding protocols, and statistical power. Crucially, current evidence remains correlational; establishing causal relationships requires integrated mechanistic investigations combining metabolomic profiling, immune marker analysis, and microbial genomics to delineate specific pathways linking gut modulation to neurological outcomes.

It is noteworthy that different strains of probiotics and types of prebiotics may exert their effects through distinct mechanisms. For example, the Lactobacillus rhamnosus GG strain may modulate intestinal immune cells to reduce the release of inflammatory cytokines (Li et al., 2020), whereas Bifidobacterium longum NCC3001 may exert direct effects on the central nervous system via the vagus nerve to enhance the synthesis of neurotransmitters such as γ-aminobutyric acid (Bercik et al., 2011). Therefore, future research should focus on elucidating the strain-specific properties and target mechanisms of various probiotics and prebiotics, as well as exploring their interactions with host genetic backgrounds, with the aim of optimizing personalized treatment strategies for ASD.

### 4.3 The role of B vitamins and vitamin D

In children with ASD, abnormalities in methylation metabolism have been widely documented, including a decreased S-adenosylmethionine to S-adenosylhomocysteine (SAM/SAH) ratio and elevated homocysteine levels (Hu et al., 2021; James et al., 2004). Studies suggest that B-vitamin supplementation may enhance methylation capacity and DNA methylation, thereby modulating the expression of genes critical for neurodevelopment (An et al., 2019; Anderson et al., 2012). Among these vitamins, vitamin B6 acts as a cofactor in the synthesis of key neurotransmitters such as gamma-aminobutyric acid (GABA) and dopamine. A deficiency in B6 may exacerbate the excitatory-inhibitory imbalance frequently observed in individuals with ASD (Wilson et al., 2021). Folate is essential for one-carbon metabolism, supporting both DNA methylation and neurotransmitter synthesis, while vitamin B12 contributes to myelin formation and the biosynthesis of monoamine neurotransmitters, collectively promoting optimal neural function (Calderón-Ospina and Nava-Mesa, 2020; Schaevitz et al., 2014). Emerging evidence indicates that insufficient maternal folate levels during pregnancy may increase the risk of ASD in offspring, whereas folate supplementation has been associated with reduced neurotoxicity, enhanced neurodevelopment, and attenuation of ASD-related symptoms (Frye et al., 2018). Similarly, vitamin B12 supplementation has been linked to improvements in communication and language abilities among children with ASD (Karhu et al., 2020). However, the efficacy of folate and B12 interventionsmay vary across individuals due to genetic polymorphisms, such as MTHFR variants—highlighting the importance of incorporating genomic profiling into personalized supplementation strategies. In addition to B vitamins, vitamin D plays a multifaceted role beyond calcium-phosphorus homeostasis, including significant contributions to neuroimmune regulation (Cui and Eyles, 2022). Vitamin D receptors are expressed in various brain regions, such as the cerebral cortex, hippocampus, and cerebellum, where they mediate neuroprotective, anti-inflammatory, and antioxidant responses (Koduah et al., 2017). Clinical studies have shown that vitamin D supplementation may lower circulating levels of pro-inflammatory cytokines, potentially mitigating neuroinflammation and improving social communication and language function in individuals with ASD (Cui et al., 2024). Future research should investigate the bidirectional interactions between vitamin D and the gut microbiota and examine its efficacy across diverse ASD subtypes.

### 4.4 Trace elements and nutritional supplementation

Children with ASD commonly exhibit elevated oxidative stress, characterized by a reduced glutathione-to-oxidized glutathione (GSH/GSSG) ratio and increased levels of malondialdehyde (MDA), a marker of lipid peroxidation (Chen et al., 2021; Efe et al., 2021). N-acetylcysteine (NAC), a precursor to glutathione, enhances intracellular antioxidant defenses and modulates the glutamatergic system, thereby alleviating repetitive behaviors and reducing irritability in children with ASD (Długosz et al., 2025; Lee et al., 2021; Song et al., 2024). Coenzyme Q10, a key component of the mitochondrial electron transport chain, is essential for ATP production and antioxidant protection. Deficiency in CoQ10 has been associated with increased neuroinflammation and cognitive dysfunction in ASD (Alhusaini et al., 2022; Mousavinejad et al., 2018). Magnesium, an important regulator of NMDA receptors within the glutamate system, plays a critical role in synaptic plasticity and neuronal signaling. Supplementation with magnesium has shown promise in improving anxiety, sleep disturbances, and attention deficits in ASD patients (Botturi et al., 2020; Patel et al., 2024). Zinc is a vital cofactor involved in both GABAergic and glutamatergic neurotransmission. Its deficiency has been linked to impaired social behavior and heightened anxiety-like symptoms in children with ASD (Alsufiani et al., 2022; Benarroch, 2023). Likewise, iron contributes to dopamine synthesis via the tyrosine hydroxylase pathway, and targeted iron supplementation has been reported to improve attention span and reduce stereotypic behaviors in ASD (Pivina et al., 2019). Moreover, omega-3 (ω-3) fatty acids, particularly eicosapentaenoic acid (EPA) and docosahexaenoic acid (DHA), are essential for neurodevelopment and synaptic function. ω-3 supplementation has been shown to attenuate neuroinflammation, regulate neurotransmitter biosynthesis, and enhance neurotrophic factor expression, ultimately improving emotional regulation and social interaction in children with ASD (Długosz et al., 2025; Walter et al., 2025).

### 4.5 The ketogenic diet

Mechanistically, the ketogenic diet (KD) works by restricting carbohydrate intake, thereby prompting the body to produce ketone bodies as an alternative energy source. This metabolic shift is hypothesized to enhance mitochondrial function, reduce oxidative stress, and modulate neurotransmitter balance (Lim et al., 2022). A handful of small-scale studies and case reports have suggested that KD may improve certain behavioral symptoms in ASD. For example, a pilot trial reported improvements in the Autism Treatment Evaluation Checklist and the Childhood Autism Rating Scale, particularly in the sociability domain, following KD intervention (Li Q. et al., 2021). Similarly, another study reported that a modified ketogenic-gluten-free diet supplemented with medium-chain triglycerides improved social affect scores on the ADOS-2 scale (Lee et al., 2018). A recent meta-analysis of randomized controlled trials (n≈338) reported a statistically significant reduction in core ASD symptoms (standardized mean difference≈–0.67) following KD interventions (Yu et al., 2022). However, the number of included KD-specific trials was limited. These findings are derived from a small number of trials, and the overall ASD sample size remains limited, which restricts the generalizability of the results. Moreover, strict adherence to KD regimens carries potential risks, including impaired growth, micronutrient deficiencies, and metabolic complications such as hypoglycemia and acidosis (Neal et al., 2008; Newmaster et al., 2022). In clinical practice, most KD studies in ASD have lacked long-term follow-up and relied on intensive metabolic monitoring, such as urinary ketone levels and blood β-hydroxybutyrate (BHB). Future research should aim to clarify the long-term effects of KD on growth, metabolic health, and gut microbiota in children with ASD, and to elucidate the underlying mechanisms. Ultimately, this could help develop safer and more individualized dietary interventions.

Personalized dietary intervention strategies will be a focal point of future research. Given the high heterogeneity of ASD, treatment strategies should be tailored to the individual needs of each patient. For instance, some children with ASD may be more sensitive to specific foods, such as gluten and casein, while others may derive greater benefits from a ketogenic diet. Future research should integrate genomics, metabolomics, and microbiomics to develop biomarker-based personalized dietary intervention protocols, thereby enhancing therapeutic efficacy while reducing potential risks (Table 1).

**TABLE 1:** Summary of nutritional and microbiota-targeted interventions in children with ASD.

Intervention	Mechanism of action	Evidence	Clinical benefits	Population	Limitations
Gluten-free casein-free diet (GFCF)	1. Eliminates gluten and casein to prevent “leaky gut”-related opioid-like peptides entering the bloodstream, potentially alleviating neurobehavioral symptoms. 2. May repair intestinal barrier and reduce inflammation, thereby modulating gut-brain axis.	1. Several small RCTs and observational studies report improvements in cognition, communication, and stereotypy. 2. Meta-analyses find no consistent benefit for core ASD symptoms.	Improvement in gastrointestinal symptoms, attention, and social behaviors in some children.	Children with GI disorders or suspected gluten/casein intolerance, or those showing signs of gut permeability or metabolic anomalies.	1. Inconsistent evidence; long-term adherence is difficult. 2. Risk of nutritional deficiencies (e.g., calcium, vitamin D) and weight loss.
Microbiota-targeted Nutrition (Probiotics/ Prebiotics/Synbiotics)	1. Modulates gut-brain axis by restoring microbial balance, reducing pro-inflammatory cytokines, and altering neurotransmitter levels. 2. Produces SCFAs to improve gut barrier and interact with vagus nerve.	1. Overall evidence from RCTs/meta-analyses shows limited statistical efficacy for core ASD symptoms. 2. Some trials show improvement in GI symptoms, irritability, or social functioning.	Alleviates GI symptoms (e.g., bloating, constipation), may help with attention, mood, and stereotyped behavior in certain subgroups.	Best for ASD children with dysbiosis or prominent GI symptoms; some evidence suggests benefits in non-GI ASD subtypes.	1. High individual variability; strain, dose, and regimen are inconsistent. 2. Limited long-term safety/effectiveness data.
Vitamin D and B supplementation	1. Vitamin D: Regulates immune and neural function, reduces inflammation, promotes serotonin synthesis. 2. B vitamins (B6, B9, B12): Involved in neurotransmitter synthesis and methylation processes. B6+Mg improves GABA synthesis and reduces excitotoxicity.	1. Vitamin D: RCTs show significant reduction in stereotypy, possible improvement in language and hyperactivity. 2. B6/Mg: Mixed results; Cochrane review found insufficient evidence.	1. Vitamin D: Reduces stereotypy, improves emotional regulation. 2. B6/Mg: May improve hyperactivity, anxiety, and sleep in some children.	Children with vitamin D deficiency, or those showing signs of metabolic or methylation abnormalities (e.g., elevated homocysteine).	1. Variable outcomes; dose needs monitoring to avoid toxicity. 2. Insufficient RCT data for B vitamins.
Antioxidants and trace nutrients (NAC, Mg, Zn, Fe, ω-3, etc.)	1. NAC: Enhances glutathione synthesis, reduces oxidative stress and glutamate excitotoxicity. 2. Magnesium: Stabilizes neuronal excitability and enhances GABAergic tone. 3. Zinc: Regulates synaptic proteins (e.g., Shank3), modulates immune function. 4. Iron: Cofactor for monoamine synthesis. 5. ω-3: Anti-inflammatory, improves synaptic plasticity.	1. NAC: Small RCTs show reduced irritability and stereotypy. 2. Mg/Zn/Fe: Observational studies suggest high prevalence of deficiency in ASD. 3. ω-3: Mixed results in improving attention or social skills.	1. NAC: Reduces aggression, irritability, and repetitive behaviors. 2. Trace elements: May help with sleep, attention, or fatigue when deficiency is present.	Children with oxidative stress, known trace element deficiencies, or specific metabolic/genetic vulnerabilities (e.g., MAOA/MTHFR variants).	1. Variable effects; lack of standardization in dosage/duration. 2. Risk of overdose/toxicity (e.g., iron overload, zinc-copper imbalance).
Ketogenic diet (KD)	1. High-fat, low-carb diet increases ketone bodies, improves mitochondrial function, and reduces ROS.—Enhances GABA synthesis, reduces neuroinflammation and mTOR signaling. 2. Alters gut microbiota toward anti-inflammatory profile.	1. Limited clinical trials show improvements in CARS, ADOS scores. 2. Significant benefit in comorbid epilepsy; animal studies show reduction in ASD-like behaviors.	Improves social interaction, reduces seizures, may benefit stereotypy and anxiety.	ASD children with comorbid epilepsy, mitochondrial dysfunction, or high neuroinflammation markers.	1. Difficult to maintain long-term; may cause nutrient imbalance or side effects (e.g., kidney stones, hyperlipidemia). 2. Requires strict medical supervision. 3. Evidence mainly from case studies or small trials.

## 5 Microbiota transplantation

Microbiota transplantation (MT), particularly fecal microbiota transplantation (FMT), involves transferring gut microbiota from healthy donors into individuals with ASD to correct dysbiosis and modulate the MGBA.

Studies have shown that FMT can significantly enhance both alpha and beta diversity of the gut microbiota (Tian et al., 2020). By restoring microbial diversity, FMT may reduce harmful bacteria such as *Escherichia coli* and Candida, while increasing beneficial species like Bifidobacterium and Lactobacillus, thereby improving gastrointestinal symptoms and reducing intestinal permeability in ASD patients (Dixit et al., 2021; Yang et al., 2023; Żebrowska et al., 2021). The potential neurobehavioral benefits of FMT are believed to involve several mechanisms: 1. Metabolic Regulation: Restoration of SCFA production, particularly butyrate, activates G-protein-coupled receptors (GPR41/43), inhibits the NF-κB signaling pathway, and reduces the release of pro-inflammatory cytokines such as IL-6 and TNF-α (Avolio et al., 2022; Siddiqui and Cresci, 2021; Su et al., 2022); 2. Gut-Brain Axis Modulation: SCFAs and serotonin precursors may influence brain function through vagal signaling or immune-mediated pathways. Animal studies have shown that FMT can upregulate brain-derived neurotrophic factor (BDNF) in the hippocampus, leading to improvements in social behavior in ASD models (D’Amato et al., 2020; Goo et al., 2020; Settanni et al., 2021; Wang et al., 2023). Although some clinical trials suggest that FMT may improve behavioral symptoms in ASD (Li N. et al., 2021; Wei et al., 2023), its clinical application still faces significant challenges. First, existing studies often involve small cohorts, short follow-up durations, and lack standardized outcome assessments.

Second, the therapeutic efficacy of FMT is highly dependent on donor microbiota quality. Currently, there is no consensus on donor selection criteria, and significant inter-donor variability exists in microbial composition and functional potential (Zheng et al., 2022). Third, the long-term safety and efficacy of FMT are still uncertain. Potential risks include immune reactions and secondary infections caused by unintended dysbiosis (Qu et al., 2022). In addition, the optimal dosage, delivery routes (e.g., oral capsules vs. colonoscopy), and factors influencing individual response to FMT remain unclear. Future multicenter, long-term studies are needed to systematically assess the impact of FMT on neurodevelopment, immune-metabolic profiles, and growth outcomes in children with ASD.

## 6 A new perspective on ASD treatment

Dysbiosis in the gut microbiota of ASD patients can lead to intestinal barrier damage, permitting harmful bacteria and metabolites to enter the bloodstream, thereby activating immune-inflammatory responses that ultimately affect neural function (Agirman et al., 2021; Brescia and Rescigno, 2021; De Angelis et al., 2015; De Angelis et al., 2015). Although short-term antibiotic treatment can suppress opportunistic pathogens and alleviate some symptoms (Wong et al., 2024), prolonged use may exacerbate microbial dysbiosis and induce antibiotic resistance. Current evidence does not support the routine use of antibiotics for ASD treatment; thus, future efforts should explore more targeted antimicrobial strategies with fewer side effects. Future research should investigate the combined use of antibiotics with probiotic or fecal microbiota transplantation (FMT) strategies to balance therapeutic efficacy and associated risks.

Vagus nerve stimulation (VNS), originally employed to treat epilepsy and depression, has increasingly attracted attention as a neuromodulatory therapy for ASD. VNS works by activating afferent vagal pathways and modulating the solitary nucleus–locus coeruleus circuitry, thereby improving the diminished parasympathetic activity observed in ASD patients (George et al., 2003; Lory et al., 2020; Zhao et al., 2025). Research indicates that VNS may enhance social interaction and emotional regulation in ASD patients by modulating the autonomic nervous system, particularly parasympathetic activity (Engineer et al., 2017). For patients unresponsive to conventional pharmacotherapy, VNS may offer a promising alternative treatment option. However, current evidence is limited by predominantly small-sample studies, and both the efficacy and adverse effects of VNS have yet to be thoroughly evaluated. VNS may lead to adverse effects, such as throat discomfort and voice changes, which may reduce its acceptability among some patients (Redgrave et al., 2018). Therefore, future studies should focus on optimizing VNS stimulation parameters, treatment protocols, and individualized application strategies to enhance therapeutic efficacy while minimizing side effects.

## 7 Conclusion and future perspectives

In recent years, research into ASD has deepened significantly, with the introduction of the MGBA providing a novel framework for understanding its pathogenesis. Gut microbiota dysbiosis is increasingly recognized as a key factor contributing to ASD, potentially influencing brain function through mechanisms such as increased intestinal permeability, altered neuroactive metabolite production, and immune system modulation. As an integrative hub of neuroendocrine, immune, and metabolic signaling, the MGBA has garnered substantial attention for its potential role in the core symptoms of ASD—including social deficits and language impairments—with important clinical implications. Despite growing interest, many key questions remain unresolved. First, the reproducibility of findings regarding gut microbiota diversity and its association with ASD symptoms is limited, often due to subject heterogeneity, small sample sizes, and inconsistent methodologies. Second, the underlying pathophysiology of ASD remains poorly understood, and the specific contribution of the MGBA warrants further exploration. Future research should integrate mechanistic studies in animal models with high-quality clinical data to clarify causal relationships between microbial dysbiosis and ASD.

In terms of treatment, MGBA-targeted interventions—including probiotics, prebiotics, and fecal microbiota transplantation—have shown promise in improving the gut environment, modulating immune-inflammatory responses, and alleviating behavioral symptoms in ASD. Although clinical outcomes remain variable, these approaches offer a foundation for more precise interventions. Long-term safety, efficacy, and patient-specific responses should be key focuses of future investigations. Additionally, emerging therapies such as VNS and selective antibiotic use may provide complementary neuromodulatory and microbiota-targeted strategies. However, both approaches remain in early stages, and their benefits and risks require further validation.

Given the high heterogeneity of ASD, precision medicine is likely to shape the future of its clinical management. Research should continue to integrate insights from genomics, microbiomics, neuroscience, and immunology through multi-omics and systems biology approaches to unravel the multifactorial nature of ASD. In particular, greater understanding of the bidirectional interactions between the gut microbiota and the central nervous system via the MGBA may lead to more effective interventions. With advancements in precision medicine, individualized treatment strategies are expected to become standard practice in ASD care. MGBA-based interventions are poised to play a critical role in improving clinical outcomes and quality of life. The integration of microbiome analysis, neuroimaging, and artificial intelligence is expected to drive the development of multidimensional therapeutic frameworks. Future research should prioritize several key areas:

1. Integrative multi-omics analysis to elucidate host–microbe interaction pathways relevant to ASD.
2. Development of targeted microbial therapies, such as engineered probiotics and phage therapy, combined with neuromodulation techniques like closed-loop VNS.
3. Large-scale randomized controlled trials to assess the long-term efficacy and safety of current and emerging interventions.
4. Early-life microbiota studies to evaluate the predictive value of prenatal and infant microbial colonization and identify critical windows for early intervention.
